# Dietary baicalin supplementation enhances growth performance in fattening Hu sheep via dual modulation of immunity and gastrointestinal microbiome-metabolic crosstalk

**DOI:** 10.1186/s40104-026-01413-y

**Published:** 2026-05-30

**Authors:** Di Ding, Yu Zhang, Haoliang Tian, Binghan Dong, Xin Wu, Shaohua Liu, Tengyun Gao, Liyang Zhang, Tong Fu

**Affiliations:** Henan International Joint Laboratory of Nutrition Regulation and Ecological Raising of Domestic Animal, College of Animal Science and Technology, Henan Agricultural University, Zhengzhou, 450046 China

**Keywords:** Baicalin, Immunity, Microbiome-metabolic crosstalk, Rumen fermentation, Sheep

## Abstract

**Background:**

Baicalin is a bioactive flavonoid from *Scutellaria baicalensis* Georgi with antioxidant, anti‑inflammatory and antibacterial properties. However, its bitter taste and susceptibility to ruminal degradation limit its practical use in mammals. Enteric coating technology might overcome these limitations by enabling targeted intestinal release. This study investigated effects of dietary supplementation with baicalin and coated baicalin on rumen fermentation, gastrointestinal microbiota, immune function and growth performance in Hu sheep.

**Results:**

Thirty-six lambs with similar body weight (33.01 ± 2.68 kg) were randomly assigned to three groups (4 replicates per group, 3 sheep per replicate). The control group was fed basal diet (CON) while treatment I (BAI) and treatment II (C-BAI) groups were fed a basal diet supplemented with 0.1% baicalin and coated baicalin, respectively. After a 60-d feeding trial, baicalin and coated baicalin supplementation improved total weight gain and average daily gain compared with CON group (*P* < 0.05). In addition, BAI and C-BAI groups exhibited higher total antioxidant capacity (*P* < 0.05) and catalase activity (*P* < 0.05) with associated lower malondialdehyde levels (*P* < 0.05). Immunoglobulin G and anti-inflammatory cytokines interleukin-4 (IL-4) were also increased (*P* < 0.001). Notably, IgM, IL-10 and IL-4 in the C-BAI group exceeded those of the BAI group (*P* < 0.001). Microbiome analysis revealed that baicalin supplementation enriched abundance of beneficial bacterial taxa including Firmicutes and *Lachnoclostridium* (*P* < 0.05) and reduced potential pathogen abundance, e.g., *Treponema* and *Ralstonia* (*P* < 0.05). The C-BAI group also showed increased abundance of the beneficial *Bradyrhizobium* compared with CON (*P* < 0.05). Metabolomic analysis revealed that baicalin altered propionate and tyrosine metabolic pathways (*P* < 0.05), while coated baicalin modulated penicillin metabolism and glyceride metabolism in jejunum (*P* < 0.05) increasing ATP production. Overall, these results indicated enhanced nutrient metabolism and gut health in the presence of dietary baicalin.

**Conclusions:**

Dietary supplementation with baicalin and coated baicalin improved growth performance, antioxidant status, immunity and beneficially modulated the microbiome-metabolome crosstalk in Hu sheep. Notably, uncoated baicalin exerted more pronounced effects on growth performance and supported a role for baicalin as a potential and functional feed additive.

**Supplementary Information:**

The online version contains supplementary material available at 10.1186/s40104-026-01413-y.

## Introduction

The growing global demand for livestock products has driven the expansion of the scale of mutton sheep farming and increased consumer awareness of animal health has intensified pressures on sheep production management [1, 2]. This underscores the urgent need for precision health strategies in sheep production. Enhancing producer resilience against disease risks is crucial for advancing sustainable and efficient ruminant production systems. Dietary antibiotic supplementation in ruminants exhibits antimicrobial and anti-inflammatory properties and enhances immune function and growth performance. However, antibiotics can induce adverse effects including gastrointestinal disturbances and critically, suppression of rumen microbial populations that compromises rumen microbiota homeostasis [3]. Recent research has demonstrated that many plants possess anti-inflammatory, antioxidant and disease prevention functions due to their distinctive biological activities [4–6]. Increasing consumer concerns regarding animal welfare and food safety has accelerated the application of medicinal plant extracts in sheep production, particularly for enhancing immunity, gastrointestinal health and disease prevention [7]. Baicalin is a leading candidate for this process since it enhances growth performance and health status in intensively reared sheep through immunomodulatory and microbiota-modulating pathways [8].

Baicalin (5,6-dihydroxy-4-oxoflav-2-en-7-yl β-D-glucopyranosiduronic acid) is derived from the roots of *Scutellaria baicalensis* (Chinese skullcap) and modulates key physiological processes via anti-inflammatory, antibacterial and microbiota-regulatory activities [9]. These include suppression of pro-inflammatory cytokine production, inhibition of bacterial protein synthesis and enhancement of intestinal barrier integrity [10]. Collectively, these lead to improved livestock health and production efficiency. The most recent examinations of baicalin effectiveness have been conducted in monogastric livestock animals to address antibiotic-induced dysfunction of the intestinal barrier that impairs long-term animal health via persistent microbial dysbiosis and immune-metabolic alterations. Baicalin has a protective effect on antibiotic-induced dysbiosis of microbial flora and enhances mitogen activated protein kinase (MAPK) signaling pathways to mitigate antibiotic-induced intestinal and microbial interference [11]. Further studies in a weaned piglet model indicated that baicalin modulated intestinal flora and reduced lincomycin-induced intestinal injury [12]. Research using ruminants has gradually increased in recent years due to the wide range of pharmacological and clinical therapeutic effects of baicalin. For instance, calf rearing remains a significant challenge and calf diarrhea accounts for 53%−63% of mortality and high disease incidence at birth [13]. Dietary baicalin supplementation was able to improve growth performance, serum antioxidant capacity and immunological function in calves while reducing the severity and incidence of diarrhea in early weaned calves [14]. These benefits are mechanistically attributed to baicalin's broad-spectrum antibacterial activity that disrupts microbial membrane integrity and suppresses metabolic pathways in diverse pathogens. Notably, this compound can reduce *Escherichia coli* viability and mitigate antibiotic resistance development in bovine mammary gland systems and is an example of its potential in managing bovine mastitis [15]. Crucially, these antimicrobial properties may exhibit unique relevance to the rumen environment where bacterial transformations could enhance baicalin's bioavailability [16]. Baicalin is also a viable antibiotic alternative since it can enhance livestock health and productivity via microbiota modulation, anti-inflammatory activities and pathogen suppression thereby providing a sustainable strategy for animal production systems.

The rumen is a specialized digestive organ in ruminants and harbors a rich microbiota that can potentially enhance baicalin efficacy via bacterial metabolic transformations [17]. Despite its considerable bioactivity and physiological benefits, the practical use of baicalin is constrained by its bitter taste and susceptibility to rumen degradation. Enteric coating technology offers a viable strategy to overcome these limitations by enabling targeted intestinal release, improving palatability, reducing gastrointestinal irritation and minimizing ruminal breakdown. Colon-targeted baicalin granules have been prepared using Eudragit S100 via a plasticizer-assisted dry powder coating technique. This approach improved the targeted delivery of baicalin to suppress its release in the upper gastrointestinal tract and mitigate ulcerative colitis [18]. Therefore, the current study was conducted to investigate the effects of dietary supplementation with baicalin and coated baicalin on rumen fermentation, the gastrointestinal microbiome, immune function and growth performance in Hu sheep. We analyzed the effects of baicalin supplementation on: (1) the growth performance and immune and antioxidant status in Hu sheep, (2) ruminal and jejunal microbiome composition and metabolite profiles, and (3) the effects of enteric coating to potentially enhance baicalin efficacy by protection from ruminal degradation to enable targeted intestinal delivery.

## Materials and methods

### Animal ethics statement

All experimental procedures and feeding management were rigorously conducted following the Guidelines for the Care and Use of Laboratory Animals established by Henan Agricultural University, according to principles of animal welfare and biosafety (HNND2025031348).

### Experimental materials

Baicalin powder (Fufeng Sinuote Biotechnology, Baoji, Shaanxi, China) was utilized as the foundational material with 85% purity. To ensure comparable intestinal exposure of bioactive baicalin between the two supplementation groups, levels were calculated based on estimated intestinal delivery rather than on an equal-product inclusion basis. The uncoated baicalin product (BAI) contained 85% baicalin. The coated baicalin product (C-BAI) consisted of 80% baicalin raw material and 20% coating material (primarily stearic acid). Based on in vitro rumen fluid incubation and subsequent calculation accounting for baicalin purity (85%), the actual rumen bypass rate of the coated product was determined to be 65%. After further correction for the baicalin content in the coated product (85% × 80% = 68%), the calculated rumen bypass rate of the active ingredient in C-BAI was 95.56% (65% ÷ 68% = 95.56%).

Accordingly, dietary inclusion rates were adjusted to achieve equivalent intestinal delivery. The BAI group received the uncoated product at 0.1% of dry matter intake (DMI), assuming negligible intestinal exposure from the uncoated form due to complete ruminal degradation. The C-BAI group received the coated product at a higher inclusion rate calculated as: C-BAI inclusion rate = (0.1% × DMI) ÷ 65%. This adjustment ensured that the estimated amount of baicalin reaching the intestinal target sites was comparable between the two treatment groups, despite their different product specifications and ruminal degradation characteristics.

### Experimental design and feeding management

Feeding experiments were conducted in an enclosed animal house in spring. The diet was formulated as a complete mixed pellet with fiber derived primarily from corn germ meal, corn bran, vinegar residue, malt root and chrysanthemum powder. Baicalin was evenly sprayed as an aqueous mist and thoroughly mixed into the diet to ensure uniform dispersion on the pellet surface. Thirty-six ram lambs (3 months old, 33.01 ± 2.68 kg body weight) were randomly allocated to three dietary treatments in a pen-based feeding system. Experimental design included four replicates per treatment with three lambs kept in each pen. Control group (CON) was provided with a basal diet while experimental groups were fed the basal diet supplemented with 0.1% powdered baicalin (BAI) and 0.1% coated baicalin (C-BAI). After a 15-d adaptation period, the 60-d trial commenced with feeding at 08:00 and 18:00 daily. The average daily baicalin intake in the BAI and C-BAI groups was approximately 1.8–1.9 g/d. This dosage level was selected to account for extensive ruminal degradation of bioactive compounds to ensure sufficient baicalin or its metabolites reached the intestinal target sites. To determine daily feed intake, any remaining feed from the prior day was weighed and recorded each morning before fresh feed was provided. Access to feed and water was provided ad libitum.

The 60 d treatment period and the pharmacological dose were chosen to maximize the detection of physiological responses and to investigate the mechanistic interactions between baicalin and the ruminant gastrointestinal ecosystem [19–21].

### Chemical analysis of diet

The diet was analyzed for nutrient composition using AOAC official methods [22]. The dry matter (DM) percentage of the diet was determined by drying the sample in an oven at 105 °C according to the method 930.15. Crude protein (CP) was measured as N × 6.25 using an automated Kjeldahl nitrogen analyzer (K9860, Jinan Haineng Instrument, Jinan, Shandong, China) in accordance with the method 948.13. Ether extract (EE) was determined by Soxhlet extraction using diethyl ether, following method 920.39. The crude ash percentage was determined by combusting the samples in a silica crucible placed in a muffle furnace according to the method 942.05. Calcium (Ca) and phosphorus (P) were quantified according to the method 968.31 and 965.17, respectively. Neutral detergent fiber (NDF) and acid detergent fiber (ADF) contents were measured via filter bag technology [23], using a semi-automatic fiber analyzer (ANKOM A200i, Ankom Technology, Macedon, NY, USA). Metabolic energy (ME) was calculated according to NRC specifications [24]. The formulation and nutritional composition of the basal diet used in this experiment are detailed in Table 1.

**Table 1:** Formulation and nutritional composition of the basal diets (dry matter basis), %

Ingredients	Content
Corn	31.50
Corn germ meal	24.50
Corn bran	14.00
Vinegar residue	18.00
Malt sprout	9.00
Chrysanthemum powder	1.00
Premix^1^	2.00
Total	100.00
Nutrient levels^2^	
Ether extract	2.66
Crude protein	14.23
Organic matter (OM)	89.47
NDF	35.67
ADF	21.41
Calcium (Ca)	1.98
Phosphorus (P)	0.45
Metabolizable energy, MJ/kg	7.15

^1^The premix provided the following per kg of diets: vitamin A, 1.5 × 10^5^ IU; vitamin D_3_, 3.5 × 10^4^ IU; vitamin E, 125 IU; nicotinic acid, 250 mg; pantothenic acid, 75 mg; biotin, 5 mg; Cu (as copper sulfate), 50 mg; Fe (as ferrous sulfate), 600 mg; Mn (as manganese sulfate), 500 mg; Zn (as zinc sulfate), 500 mg; Se (as sodium selenite), 5 mg; Co (as cobalt sulfate), 5 mg

^2^ Nutritional levels were measured values, except that metabolizable energy was calculated

### Blood collection

On the final day of the experiment, six sheep per group were randomly selected for blood sampling. Blood samples (10 mL) were collected from the jugular vein from fasted sheep. The samples were allowed to clot for 45 min at room temperature. After centrifugation at 4 °C, 1,538 × *g*, the supernatant was collected, transferred into a centrifuge tube and stored at −20 °C for the determination of antioxidant and immunological parameters.

### Detection of serum antioxidant, immune indexes and inflammatory factors

Serum concentrations of malondialdehyde (MDA) and total antioxidant capacity (T-AOC) along with activities of catalase (CAT) and glutathione peroxidase (GPx) were quantified colorimetrically using commercial kits and a microplate reader (RT-6100, Rayto Life Science Corp, Shenzhen, Guangdong, China). Immunoglobulin (Ig) profiles of IgA, IgG and IgM as well as cytokine levels comprising interleukin (IL)-1β, IL-4, IL-6, IL-10, tumor necrosis factor-α, and interferon-γ were determined by enzyme-linked immunosorbent assay with commercial kits (Jiangsu Meimian Industrial, Yancheng, Jiangsu, China) following the manufacturer protocols.

### Rumen fluid and contents collection

After blood sampling, rumen fluid samples were collected from the same six sheep per group using an oral stomach tube. Rumen fluid pH was immediately measured in triplicate with a calibrated pH meter (pHS-3C, Shanghai LeiMag Instrument, Shanghai, China) and filtered through four-layer sterile gauze (100 μm pore size). Aliquots for volatile fatty acid (VFA) analysis were stored at −20 °C. Subsequently, animals underwent 24 h fasting with 2 h water deprivation for the collection of rumen and intestinal contents. After slaughter, jejunal digesta were collected from the same six sheep per group. The reticulo-rumen and jejunum were immediately isolated. Rumen digesta and jejunal contents were collected using RNA-free spatulas, placed in a polyethylene freezer tube, snap-frozen in liquid N_2_, and stored at −80 °C for microorganisms and metabolites. Metabolomic analysis and microbiome analysis was performed on all 18 ruminal samples and 18 jejunal samples.

### Determination of rumen fermentation parameters

Thawed rumen fluid aliquots (4 °C, 12 h) were centrifuged at 10,956 × *g* for 15 min (4 °C). Ammonia-nitrogen (NH_3_-N) concentration was quantified by phenol-hypochlorite colorimetry using a microplate reader (RT-6100, Rayto Life Science Corp, Shenzhen, Guangdong, China) [25]. VFA included acetic acid (C₂), propionic acid (C₃), butyric acid (C₄), isovaleric acid (iC₅), and valeric acid (C₅) that were separated via gas–liquid chromatography (TRACE 1310, Thermo Fisher Scientific, Waltham, MA, USA) equipped with a polar capillary column [26].

### 16S rDNA sequencing and metabolomics determination of rumen and jejunal contents

Rumen and jejunal digesta samples were sent to Guangzhou Gene Denovo Biotechnology Co., Ltd. (Guangzhou, Guangdong, China) for microbial community analysis using 16S rDNA sequencing. The V3–V4 region of bacterial 16S rRNA was amplified using primers 341 F (5′-ACTCCTACGGGAGGCAGCA-3′) and 806R (5′-GGACTACHVGGGTWTCTAAT-3′) and the sequencing library was established and sequenced by Illumina Miseq PE300. Aliquots (50 mg) of thawed rumen and jejunal digesta were homogenized in 2 mL polypropylene tubes containing 1,000 μL ice-cold methanol by vortex mixing (1 min, 4 °C). After centrifugation at 15,753 × *g* for 10 min at 4 °C, 450 μL of the supernatant was transferred to a new tube and mixed with 150 μL of an internal standard solution consisting of 2-chlorobenzylalanine at 4 μg/mL in 80% (v/v) methanol. The mixture was then filtered through 0.22-μm polyvinylidene fluoride (PVDF) membranes to obtain samples ready for LC–MS analysis. Metabolites were resolved using a Thermo Ultimate 3000 UHPLC system (Waltham, MA, USA) equipped with an ACQUITY UPLC HSS T3 column (150 mm × 2.1 mm, 1.8 μm; Waters, Milford, MA, USA) maintained at 40 °C with a 0.25 mL/min flow rate. Gradient elution of analytes was performed with 0.1% formic acid aqueous solution and 0.1% formic acid acetonitrile solution or 5 mmol/L ammonium formate aqueous solution and acetonitrile solution at a flow rate of 0.25 mL/min.

### Determination of growth performance

At the start of the experiment, the sheep were fasted for 12 h before initial body weight (IBW) was measured individually. Throughout the trial, daily feed intake was recorded and final body weight (FBW) was measured on d 60. These data were used to calculate total weight gain (TWG), average daily gain (ADG) and feed conversion ratio (F/G).

### Statistical analysis

All data were preprocessed using Microsoft Excel 2019 and were expressed as means together with the standard error of the mean (SEM). For growth performance data (e.g., feed intake, ADG, FCR), the pen was considered the experimental unit, with 4 replicate pens per group (*n* = 4). For serum parameters, rumen fermentation, and omics data, samples were collected from individual animals (*n* = 6 per group). Statistical analyses were performed using SPSS 27.0 (IBM SPSS Statistics, Version 27.0, IBM, Armonk, NY, USA). Significant differences among groups were determined by one-way analysis of variance (ANOVA) followed by Duncan's multiple range test for post hoc comparisons. Differences were considered statistically significant at *P* < 0.05, and distinct superscript letters were used to indicate significant differences between group means.

The mathematical model for the ANOVA was: *X*_*ij*_ = *μ* + *α*_*i*_ + *ε*_*ij*_, where *X*_*ij*_ denoted the *j*-th observation under the *i*-th treatment group, *μ* denoted the population mean, *α*_*i*_ denoted the fixed effect of the *i*-th treatment (*i* = 1, 2, …, *k*), *ε*_*ij*_ denoted the random experimental error associated with the *j*-th observation in the *i*-th group. The *ε*_*ij*_ were assumed to be independently and identically distributed as normal random variables with a mean of zero and a constant variance *σ*^2^ (*ε*_*ij*_ ~ N (0, σ^2^)).

## Results

### Effects of baicalin and coated baicalin on growth performance of Hu sheep

Across our experimental animals including the controls, there were no differences in the initial body weights (*P* = 0.757). In contrast, the final body weights were higher in the BAI compared with the CON group (*P* = 0.029). Consequently, compared to the CON group, both BAI and C-BAI treatment improved total weight gain (*P* < 0.001). Additionally, the feed conversion ratio decreased by 16.1% in the BAI group (*P* < 0.05) indicating an increased efficiency, and the C-BAI group exhibited an upward trend compared to the CON group (Table 2). These results indicated that dietary baicalin supplementation improved the growth performance of Hu sheep over the 60 d experimental period.

**Table 2:** Effects of baicalin and coated baicalin on growth performance of Hu sheep (*n* = 4)

Items	Treatments			SEM	*P*-value
	CON	BAI	C-BAI		
IBW, kg	33.50	33.08	32.83	0.355	0.757
FBW, kg	46.76^b^	49.85^a^	48.64^ab^	0.490	0.029
TWG, kg	13.04^b^	16.77^a^	15.82^a^	0.465	< 0.001
DMI, g/d	1,660.50^b^	1,820.99^ab^	1,902.47^a^	35.709	0.005
ADG, g/d	217.27^b^	279.44^a^	263.64^a^	7.753	< 0.001
F/G	7.82^a^	6.56^b^	7.52^ab^	0.232	0.020

*CON* Basal diet, *BAI* Basal diet with 0.1% baicalin, *C-BAI* Basal diet with 0.1% coated baicalin, *IBW* Initial body weight, *FBW* Final body weight, *TWG* Total weight gain, *DMI* Dry matter intake, *ADG* Average daily gain, *F/G* Feed conversion ratio

Results are expressed as means. Within a row, mean values labeled with different lowercase superscript letters differ statistically (*P* < 0.05), whereas values marked with the same letter or lacking superscripts show no significant difference (*P* > 0.05)

### Effects of baicalin and coated baicalin on antioxidant, immune and anti-inflammatory ability of Hu sheep

Dietary supplementation with BAI or C-BAI significantly decreased MDA levels (*P* = 0.004) indicative of an effective anti-oxidative response. Furthermore, both the BAI and C-BAI groups enhanced activity of CAT and T-AOC relative to the CON group (*P* < 0.05). Although the activity of GSH-Px did not differ significantly among the groups, BAI group increased by 8.9% and C-BAI group increased by 4.9% compared to CON (*P* = 0.217) and indicated a consistent upward trend (Table 3). Collectively, the BAI and C-BAI groups displayed indications of alleviated oxidative stress.

**Table 3:** Effects of baicalin and coated baicalin on serum antioxidant capacity of Hu sheep (*n* = 6)

Items	Treatments			SEM	*P*-value
	CON	BAI	C-BAI		
MDA, nmol/mL	3.34^a^	2.21^b^	2.57^b^	0.158	0.004
CAT, U/mL	9.35^b^	12.60^a^	11.63^a^	0.541	0.030
T-AOC, U/mL	4.26^b^	5.81^a^	6.00^a^	0.237	< 0.001
GSH-Px, nmol/mL	393.45	428.36	412.72	8.049	0.217

*CON* Basal diet, *BAI* Basal diet with 0.1% baicalin, *C-BAI* Basal diet with 0.1% coated baicalin, *MDA* Malondialdehyde, *CAT* Catalase, *T-AOC* Total antioxidant capacity, *GSH-Px* Glutathione peroxidase

Results are expressed as means. Within a row, mean values labeled with different lowercase superscript letters differ statistically (*P* < 0.05), whereas values marked with the same letter or lacking superscripts show no significant difference (*P* > 0.05)

In addition to these effects on oxidative response in the sheep, serum IgG levels significantly exceeded CON by 10.3% (*P* < 0.001) in both BAI and C-BAI groups. Notably, IgA and IgM levels were also higher in C-BAI than in both BAI and CON (*P* < 0.05). Concurrently, pro-inflammatory cytokines were suppressed and TNF-α was significantly decreased in BAI and C-BAI versus CON (*P* < 0.001) and IL-6 followed the same pattern (*P* < 0.001). Anti-inflammatory mediators such as IL-4 were at their greatest levels in the C-BAI group versus BAI and CON (*P* < 0.001). IL-10 increased exclusively in C-BAI and surpassed both CON and BAI (*P* < 0.001) (Table 4).

**Table 4:** Effects of baicalin and coated baicalin on serum immune and anti-inflammatory ability of Hu sheep (*n* = 6)

Items	Treatments			SEM	*P*-value
	CON	BAI	C-BAI		
IgA, μg/mL	182.05^b^	200.90^b^	241.87^a^	7.334	0.002
IgG, μg/mL	43.84^b^	55.83^a^	61.29^a^	1.794	< 0.001
IgM, μg/mL	1,375.61^c^	1,740.02^b^	1980.09^a^	56.750	< 0.001
TNF-α, pg/mL	128.18^a^	99.38^b^	92.46^b^	4.269	< 0.001
IFN-γ, pg/mL	463.05	434.14	465.10	14.229	0.624
IL-1, pg/mL	527.88	531.86	581.42	21.548	0.322
IL-4, pg/mL	30.43^c^	41.69^b^	51.56^a^	1.707	< 0.001
IL-6, pg/mL	133.01^a^	107.74^b^	101.84^b^	3.634	< 0.001
IL-10, pg/mL	86.09^b^	88.87^b^	118.90^a^	3.295	< 0.001

*CON* Basal diet, *BAI* Basal diet with 0.1% baicalin, *C-BAI* Basal diet with 0.1% coated baicalin, *Ig* Immunoglobulin, *TNF*-α Tumour necrosis factor-α, *IFN*-γ Interferon-γ, *IL* Interleukin

Results are expressed as means. Within a row, mean values labeled with different lowercase superscript letters differ statistically (*P* < 0.05), whereas values marked with the same letter or lacking superscripts show no significant difference (*P* > 0.05)

### Effects of baicalin and coated baicalin on rumen fermentation characteristics of Hu sheep

Rumen fluid pH, NH_3_-N and acetic acid levels were unaffected by baicalin supplementation (*P* > 0.05). Propionic acid levels were greater in BAI versus CON and C-BAI (*P* = 0.029) leading to a lower acetate-to-propionate (A/P) ratio in BAI than that in the other two groups (*P* < 0.05). Therefore, baicalin dietary supplementation altered the VFA production in the rumen of these sheep (Table 5).

**Table 5:** Effects of baicalin and coated baicalin on rumen fermentation characteristics of Hu sheep (*n* = 6)

Items	Treatments			SEM	*P*-value
	CON	BAI	C-BAI		
pH	5.98	5.79	5.92	0.074	0.568
NH_3_-N, mg/dL	24.11	34.88	30.52	2.527	0.244
Total VFA, mmol/L	89.44	100.90	91.17	8.840	0.864
Acetic acid, mmol/L	55.37	56.35	50.21	3.996	0.837
Propionic acid, mmol/L	16.82^b^	24.32^a^	17.22^b^	1.442	0.029
Butyric acid, mmol/L	12.34	15.81	12.54	2.161	0.802
Valeric acid, mmol/L	0.68	0.64	0.55	0.088	0.858
Isovaleric acid, mmol/L	1.09	1.99	1.69	0.186	0.125
A/P	2.93^a^	2.12^b^	2.30^a^	0.137	0.049

*CON* Basal diet, *BAI* Basal diet with 0.1% baicalin, *C-BAI* Basal diet with 0.1% coated baicalin, *VFA* Volatile fatty acids, *A/P* Acetic/Propionic acid ratio

Results are expressed as means. Within a row, mean values labeled with different lowercase superscript letters differ statistically (*P* < 0.05), whereas values marked with the same letter or lacking superscripts show no significant difference (*P* > 0.05)

### Effects of baicalin and coated baicalin on microbial diversity and composition in rumen and jejunum of Hu sheep

**Fig. 1:**
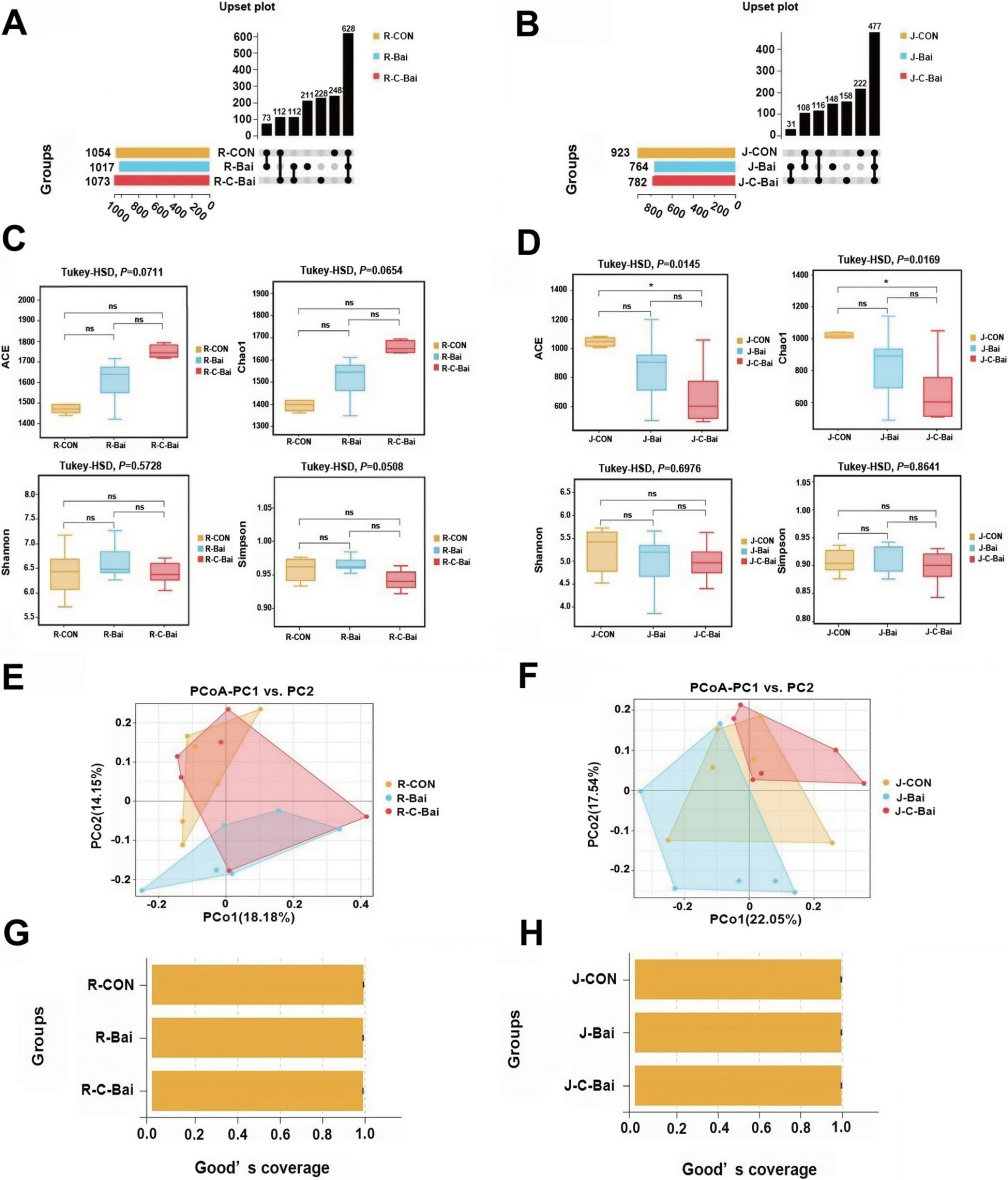
Effects of baicalin and coated baicalin on OTU level upset and rumen and jejunum microbiota of Hu sheep. **A** Effects of baicalin and coated baicalin on upset plots of OTU levels in the rumen of Hu sheep. **B** Effects of baicalin and coated baicalin on upset plots of OTU levels in the jejunum of Hu sheep. **C** Effect of baicalin and coated baicalin on the α diversity of the rumen microbiota of Hu sheep. **D** Effect of baicalin and coated baicalin on the α diversity of the jejunum microbiota of Hu sheep. **E** Effect of baicalin and coated baicalin on the β diversity of the rumen microbiota of Hu sheep. **F** Effect of baicalin and coated baicalin on the β diversity of the jejunum microbiota of Hu sheep. **G** Denotes the microbial Goods _coverage index in the rumen of Hu sheep. **H** Denotes the microbial Goods _ coverage index in the jejunum of Hu sheep. *n* = 6. ^ns^*P* > 0.05, ^*^P < 0.05

Analysis of 16S rRNA sequences identified 628 core OTUs shared across CON, BAI and C-BAI groups (Fig. 1A). Pairwise comparisons demonstrated distinct overlapping patterns where CON and C-BAI shared 112 OTUs and BAI and C-BAI also shared 112 OTUs. In contrast, CON and BAI shared 73 OTUs. For reference, the OTU counts for the CON, BAI, and C-BAI groups were 1,054, 1,017 and 1,073, respectively. Jejunal sequencing identified 477 core OTUs and CON and C-BAI shared 116 OTUs (Fig. 1B). CON and BAI shared 108 OTUs and BAI and C-BAI group shared 31 OTUs. The precise OTUs for CON, BAI and C-BAI were 923, 764 and 782, respectively. Baicalin did not affect ruminal microbial richness (ACE and Chao1; *P* > 0.05) or sample diversity (Shannon and Simpson; *P* > 0.05) (Fig. 1C). In contrast, C-BAI reduced jejunal species richness compared with the CON group (ACE and Chao1; *P* < 0.05) while diversity (Shannon and Simpson) was maintained (Fig. 1D). Principal coordinate analysis (PCoA) based on Bray–Curtis dissimilarity revealed spatial structuring of microbial communities (Fig. 1E and F). Distance between samples in different groups are directly proportional to microbial community composition similarities.

### Effects of baicalin and coated baicalin on the main microflora in rumen and jejunum of Hu sheep

**Fig. 2:**
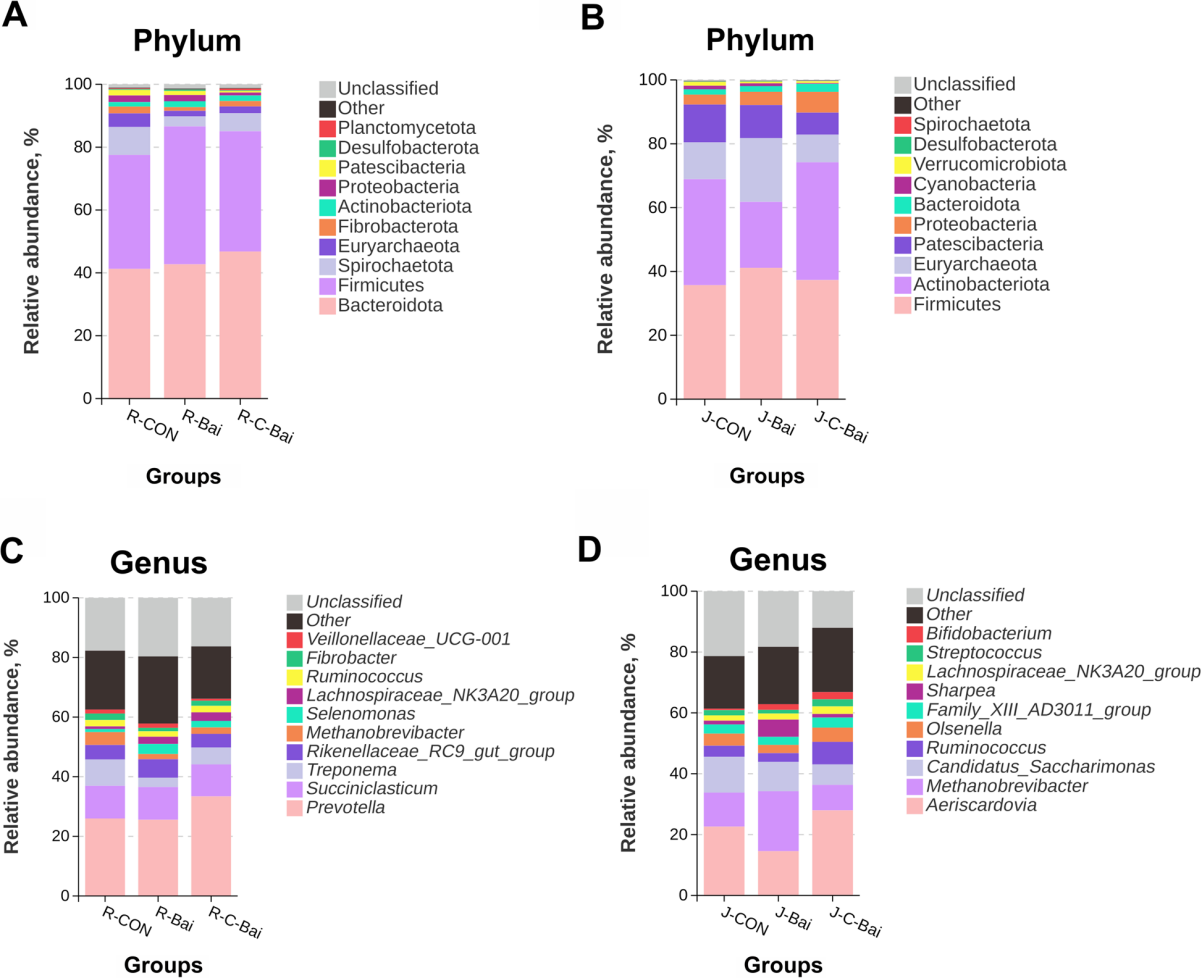
Effects of baicalin and coated baicalin on the rumen and jejunum microbiota of Hu sheep. **A** Effects of baicalin and coated baicalin on the phylum level of rumen microbiota in Hu sheep. **B** Effects of baicalin and coated baicalin on the phylum level of jejunum microbiota in Hu sheep. **C** Effects of baicalin and coated baicalin on the genus level of rumen microbiota in Hu sheep. **D** Effects of baicalin and coated baicalin on the genus level of jejunum microbiota in Hu sheep. *n* = 6

The taxa identified included Bacteroidota (41.25% to 46.79%) and Firmicutes (36.28% to 43.91%) as the dominant ruminal phyla (Fig. 2A). In the jejunal microbiota, Firmicutes (35.70% to 41.13%) and Actinobacteriota (20.67% to 36.91%) collectively represented 68.91% to 78.04% of sequences (Fig. 2B). At the genus level, ruminal *Prevotella* (25.55% to 33.38%) and *Succiniclasticum* (10.74% to 10.99%) together exceeded 36% relative abundance (Fig. 2C). Jejunal microbiota was dominated by *Aeriscardovia* (14.55% to 27.96%) and *Methanobrevibacter* (8.38% to 19.70%) (Fig. 2D).

**Fig. 3:**
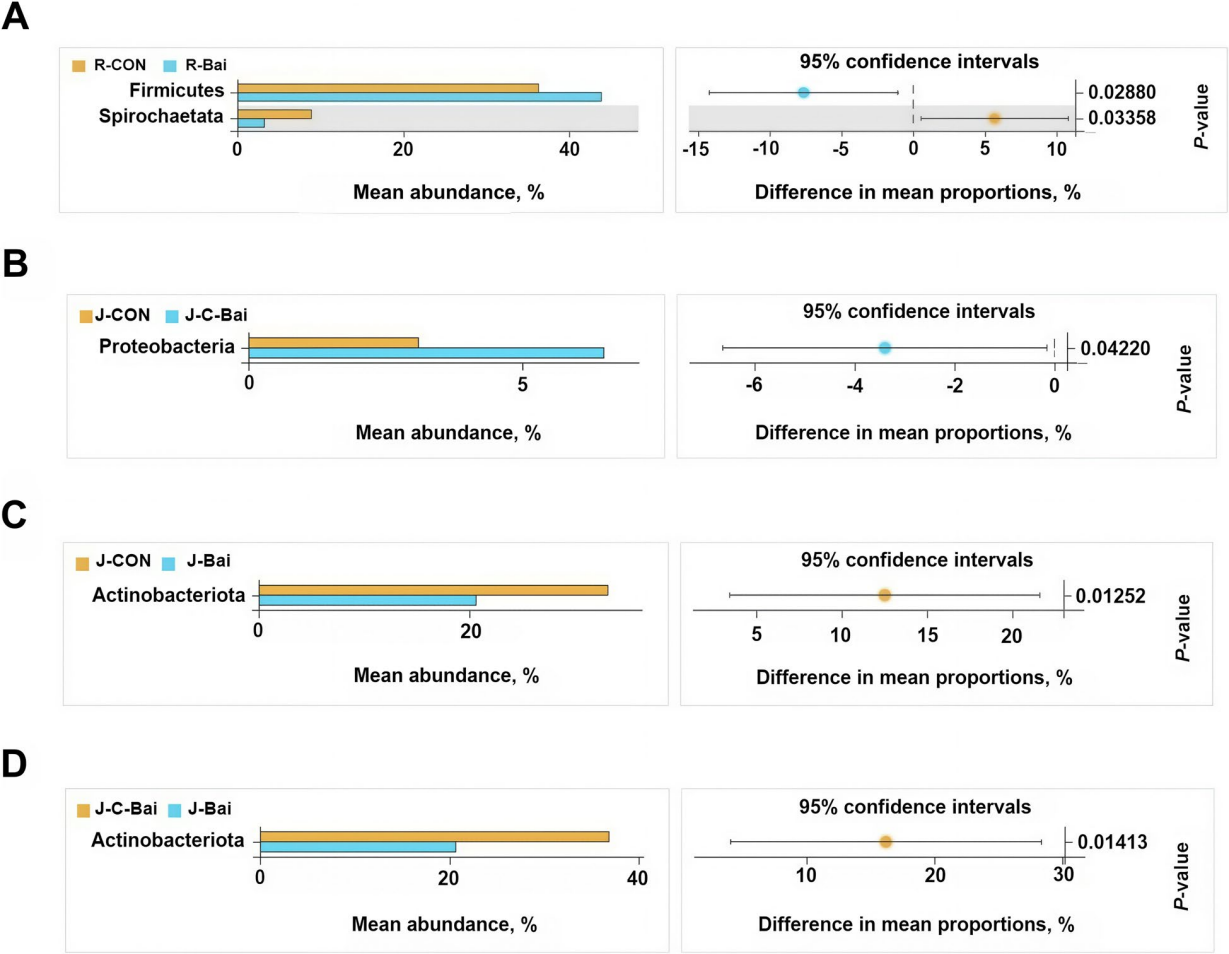
Differences in the ruminal and jejunal microbiota at the phylum level of Hu sheep. **A** Differences in the ruminal microbiota at the phylum level between the CON and BAI groups in Hu sheep. **B** Difference in the jejunal microbiota at the phylum level between the CON group and C-BAI group in Hu sheep. **C** Difference in the jejunal microbiota at the phylum level between the CON group and BAI group in Hu sheep. **D** Difference in the jejunal microbiota at the phylum level between the C-BAI group and BAI group in Hu sheep. *n* = 6

**Fig. 4:**
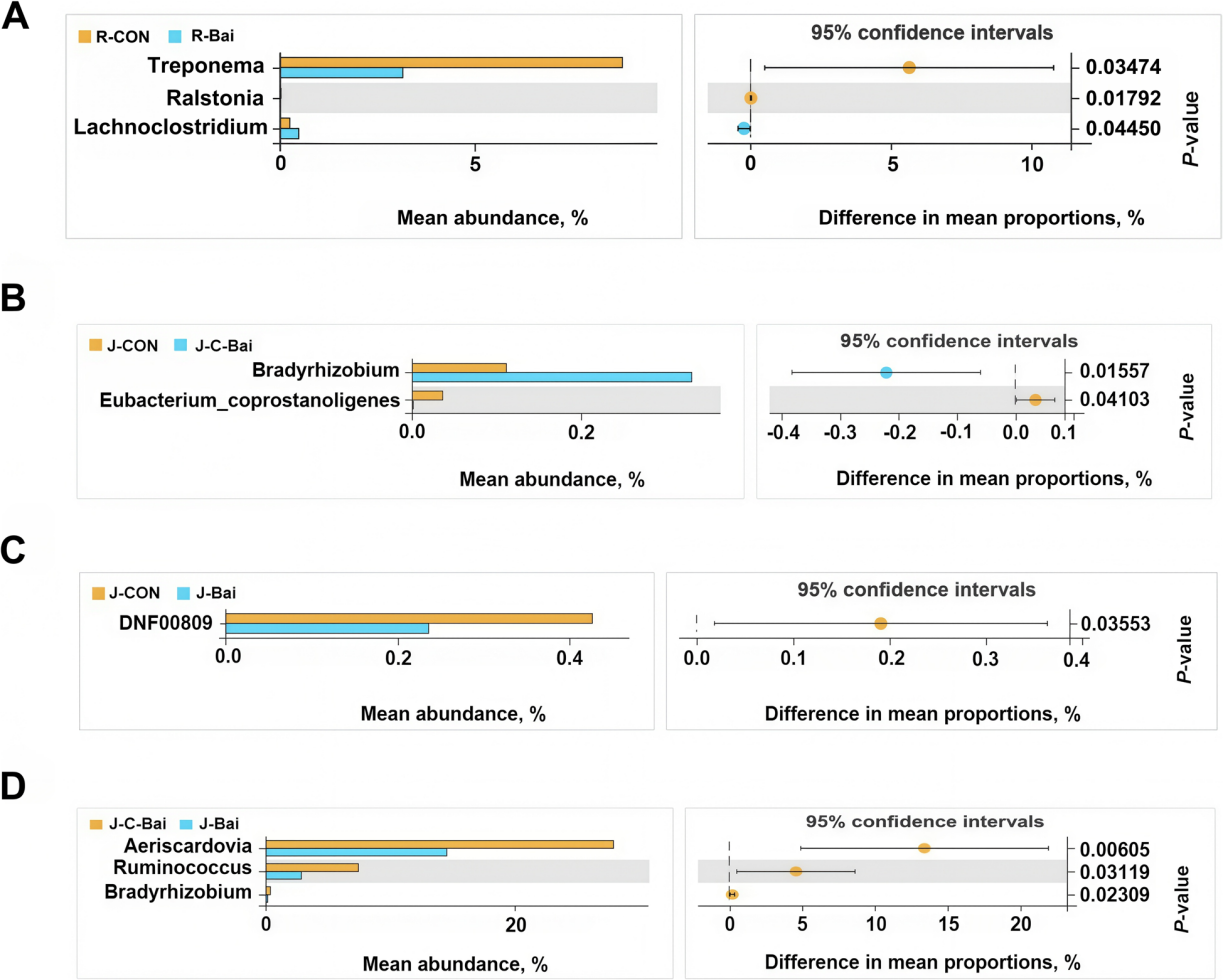
Differences in the ruminal and jejunal microbiota at the genus level of Hu sheep. **A** Differences in the ruminal microbiota at the genus level between the CON and BAI groups in Hu sheep. **B** Differences in the jejunal microbiota at the genus level between the CON group and C-BAI group in Hu sheep. **C** Difference in the jejunal microbiota at the genus level between the CON group and BAI group in Hu sheep. **D** Differences in the jejunal microbiota at the genus level between the C-BAI group and BAI group in Hu sheep. *n* = 6

In order to further analyze the differences in bacterial composition between the three groups at the phylum and genus levels, Welch's *t*-test was used. At the rumen phylum level, compared with the CON group, the BAI group showed significantly increased, Firmicutes abundance (*P* < 0.05) and reduced Spirochaetota abundance (*P* < 0.05) (Fig. 3A). Proteobacteria relative abundance was higher in the C-BAI group than CON (*P* < 0.05, Fig. 3B). The Actinobacteriota relative abundance in the jejunum was lower in the BAI group (*P* < 0.05) than in the CON and C-BAI groups (*P* < 0.05) (Fig. 3C and D). At the genus level in the rumen, the BAI group exhibited a significantly higher relative abundance of *Lachnoclostridium* (*P* < 0.05) but lower relative abundances of *Treponema* and *Ralstonia* (*P* < 0.05) than the CON group (Fig. 4A). In the jejunum, *Bradyrhizobium* abundance in the C-BAI group was elevated compared to the CON group (*P* < 0.05) while *Eubacterium coprostanoligenes* was reduced (*P* < 0.05, Fig. 4B). Concurrently, the BAI group showed a significantly reduced relative abundance of DNF00809 compared with CON (*P* < 0.05, Fig. 4C). Furthermore, the C-BAI group demonstrated significantly higher relative abundances of *Aeriscardovia, Ruminococcus* and *Bradyrhizobium* than the BAI group (*P* < 0.05, Fig. 4D).

### Correlation analysis between rumen volatile fatty acid concentrations and rumen flora

**Fig. 5:**
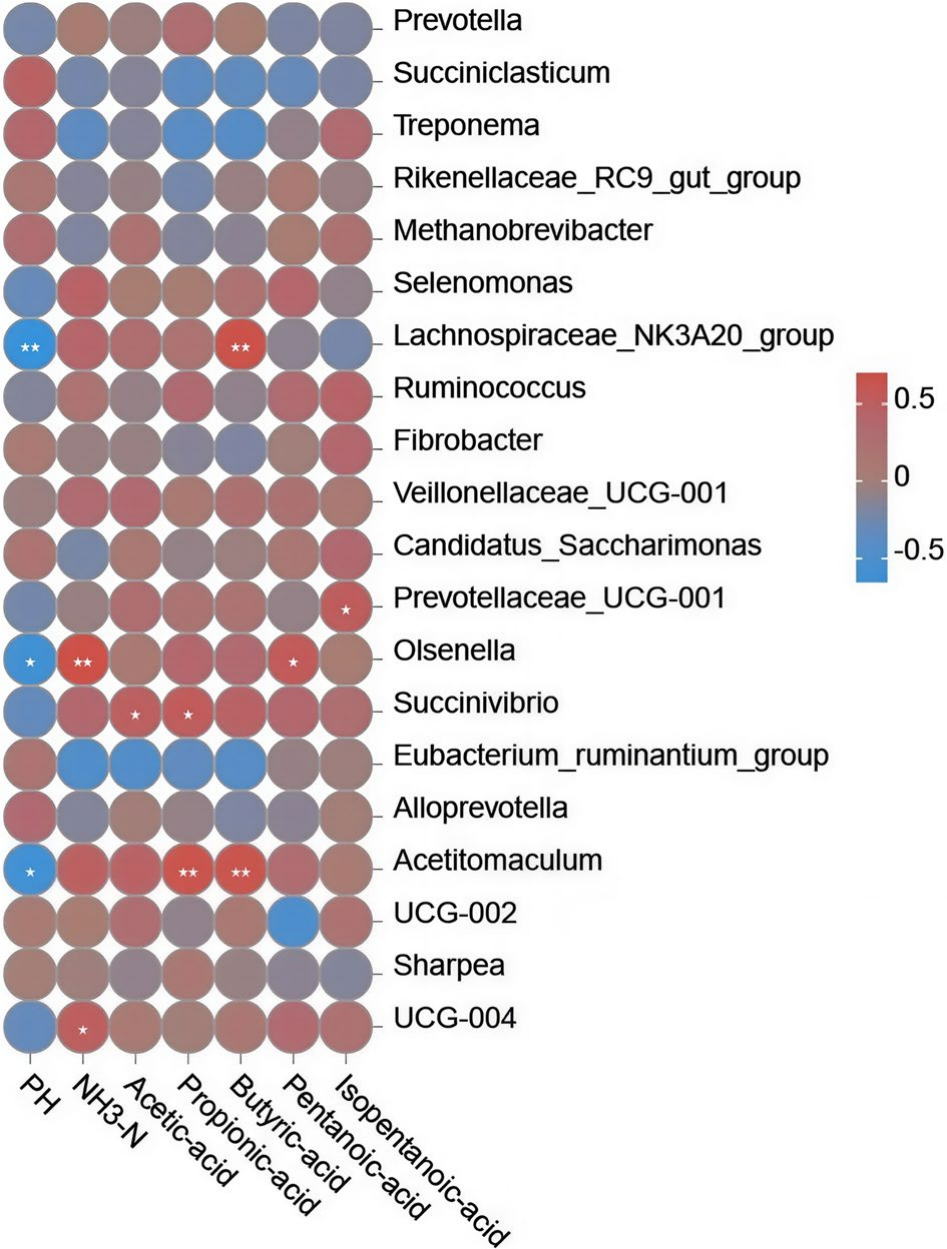
Heat map of correlation between rumen volatile fatty acids and microbial diversity and abundance. ^*^P < 0.05, ^**^P < 0.01, ^***^P < 0.001

We next examined whether there were correlations between rumen chemical conditions. We found that pH was negatively correlated with *Olsenella* and *Acetitomaculum* abundance (both *P* < 0.05) and *Lachnospiraceae_NK3A20_group* (*P* < 0.01). NH_3_-N levels displayed significant positive correlations with the abundance of *UCG-004* (*P* < 0.05) and *Olsenella* (*P* < 0.01). Acetic acid levels were positively correlated with the relative abundance of *Succinivibrio* (*P* < 0.05). Propionic acid concentration positively correlated with *Acetitomaculum* (*P* < 0.01) and *Succinivibrio* relative abundance (*P* < 0.05). Additionally, butyric acid levels were positively correlated with *Acetitomaculum* abundance (*P* < 0.01). A positive correlation was also found between *Olsenella* and valeric acid levels (*P* < 0.05). Additionally, isovaleric acid levels were correlated with *Prevotellaceae_UCG-001* abundance (*P* < 0.05; Fig. 5).

### Effects of baicalin and coated baicalin on rumen and jejunal metabolite profiles and metabolic pathways in Hu sheep

**Fig. 6:**
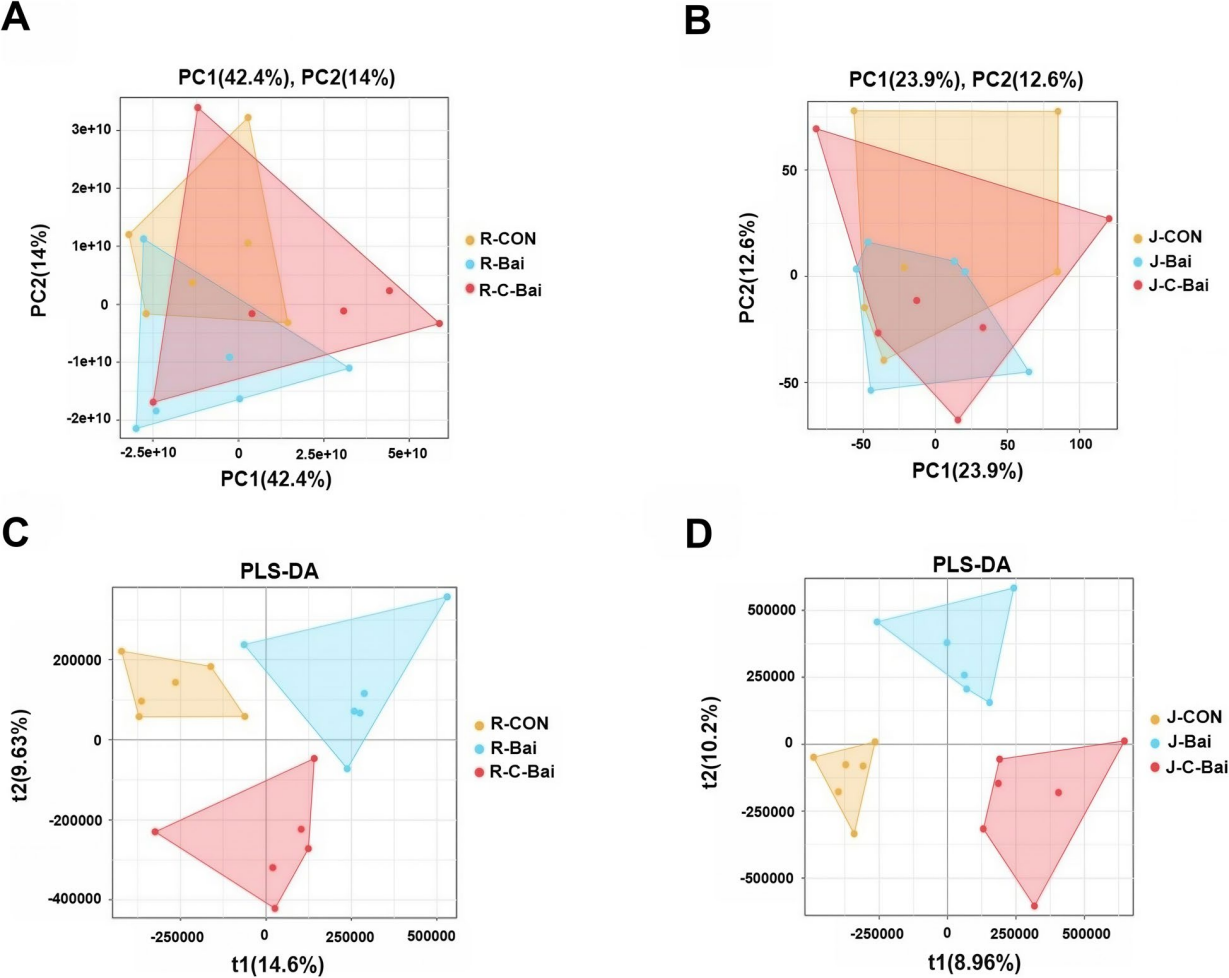
Effects of baicalin and coated baicalin on PCA and PLS-DA of ruminal and jejunal contents of Hu sheep. **A** Effects of baicalin and coated baicalin on PCA of ruminal contents of Hu sheep. **B** Effects of baicalin and coated baicalin on PCA of jejunum contents of Hu sheep. **C** Effects of baicalin and coated baicalin on PLS-DA of ruminal contents of Hu sheep. **D** Effects of baicalin and coated baicalin on PLS-DA of jejunal contents of Hu sheep

**Fig. 7:**
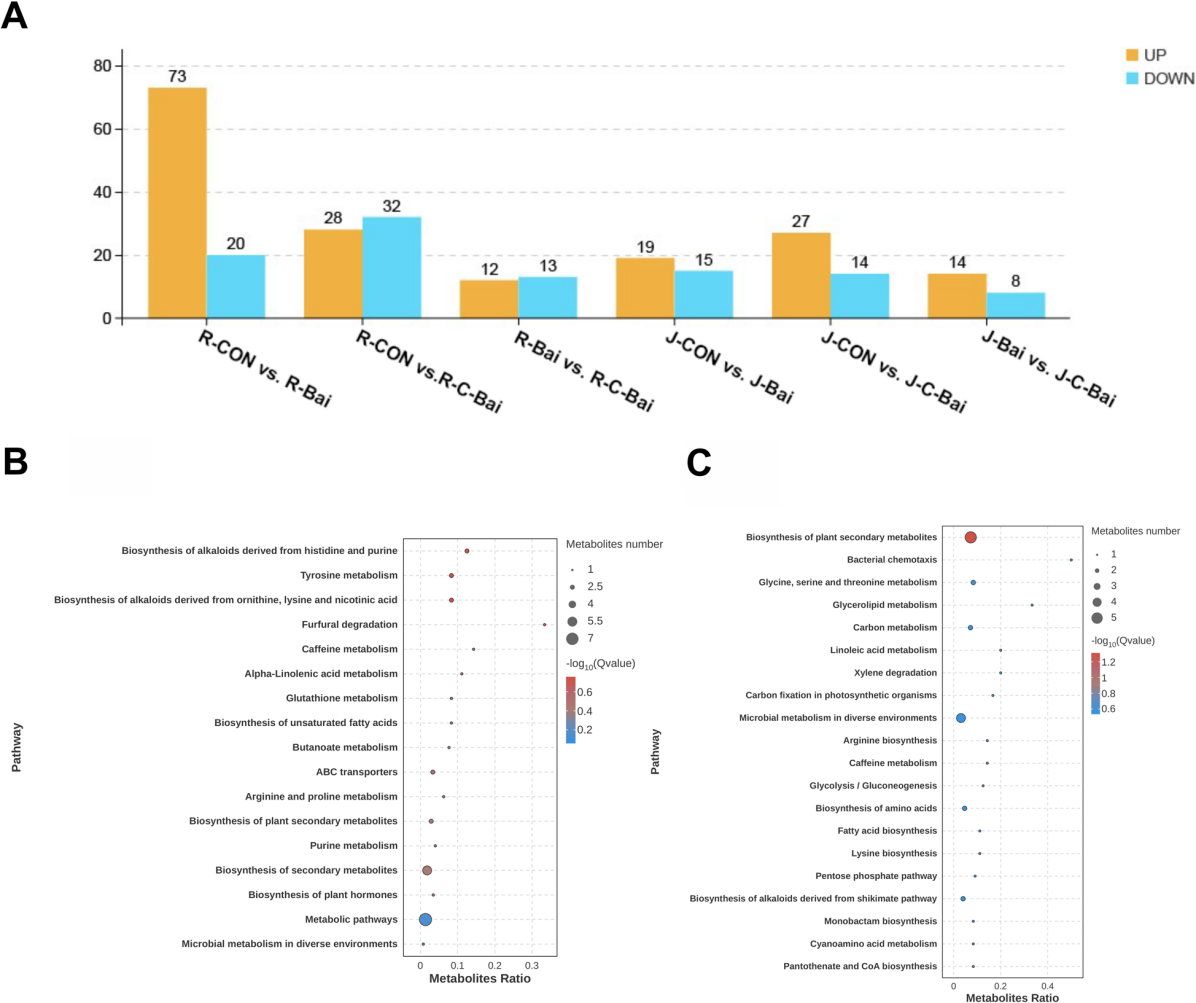
Effects of baicalin and coated baicalin on metabolites and metabolic pathways in the rumen and jejunum of Hu sheep. **A** Effects of baicalin and coated baicalin on metabolites in the rumen and jejunum of Hu sheep. **B** KEGG enrichment analysis of the first 20 pathways of differential metabolites in rumen of Hu sheep in CON group and BAI group. **C** KEGG enrichment analysis of the first 20 pathways of differential metabolites in jejunum of Hu sheep in CON group and C-BAI group. *n* = 6

Differential metabolite and metabolic pathway analyses were conducted on ruminal and jejunal contents from the three groups to research impacts of coated baicalin and baicalin in Hu sheep. Principal component analysis (PCA) revealed no distinct separation among the groups (Fig. 6A and B). Orthogonal projections to latent structures-discriminant analysis (OPLS-DA) segregated the CON, BAI and C-BAI groups in ruminal and jejunal contents (Fig. 6C and D). Differential metabolites were identified via PLS-DA (VIP > 1; FDR < 0.05). Rumen analysis identified 93 differential abundant metabolites between CON and BAI groups (Fig. 7A) with 73 metabolites significantly upregulated and 20 downregulated in the BAI group compared with the CON group. Similarly, jejunal analysis revealed that 34 differential metabolites (19 upregulated and 15 downregulated) in the BAI group compared to the CON group. Jejunal samples from the CON and C-BAI groups consisted of 41 differential metabolites (27 upregulated and 14 downregulated). Additionally, 22 differential metabolites that distinguished C-BAI from BAI group exhibited 14 upregulated and 8 downregulated metabolites in C-BAI relative to the BAI group (Tables S1–S3).

KEGG enrichment analysis identified 30 significantly altered metabolic pathways in rumen between CON and BAI groups. In the jejunum, 23 altered pathways were enriched between CON and C-BAI groups while 6 differential pathways distinguished the BAI and C-BAI groups. Dietary baicalin supplementation modulated key ruminal pathways, notably propionate (*P* = 0.023) and tyrosine metabolism (*P* = 0.025) (Fig. 7B, Table S4). C-BAI supplementation influenced jejunal pathways including penicillin metabolism (*P* = 0.015), parenteral cephalosporin metabolism (*P* = 0.015) and glyceride metabolism (*P* = 0.045) (Fig. 7C, Table S5). Further analysis identified critical pathways enriched for carbohydrate and protein metabolism: Glycolysis (map00010), Pentose phosphate pathway (map00030), Arginine biosynthesis (map00220) and Glycine-serine-threonine metabolism (map00260) (Fig. 7C).

## Discussion

Growth performance is a primary indicator of animal growth. Previous studies have indicated that dietary flavonoid supplementation enhances growth performance by improving intestinal health and increasing feed intake [27]. Specifically**,** dietary baicalin modulated the gut microbiota and the PI3K-Akt signaling pathway that in turn, enhanced appetite and increased isovaleric acid levels leading to improved body weight gains in mice [28]. In piglets, *E. coli* infection caused intestinal inflammation leading to impaired growth, whereas the addition of baicalin restored intestinal health and improved feed efficiency and body weight [29]. Similarly, in our experiments, baicalin increased dry matter intake and average daily gain and elevated propionic acid levels leading to positive effects on the Hu sheep. Thus, the impact of dietary baicalin supplementation on growth performance may vary across different animal species.

An oxidative-antioxidant imbalance develops in animals exposed to pathological conditions or environmental stressors such as hyperthermia, dehydration and nutritional deficiency. This imbalance arises when impaired scavenging systems, primarily the enzymatic antioxidants CAT, GSH-Px and T-AOC fail to neutralize excessive free radicals and this leads to cellular damage. MDA is a key biomarker of oxidative stress that is produced during enzymatic and non-enzymatic peroxidation of fatty acids [30]. Reactive oxygen species (ROS) overwhelm endogenous antioxidant defenses under excessive production, causing oxidative stress that damages cellular components. In previous studies, activation of the Nrf2 pathway induced antioxidant responses and baicalin attenuated oxidative stress by activating Nrf2. This process upregulated protective enzymes including glutathione S-transferase and glutathione peroxidase 1 [31]. Notably, supplementation with either baicalin or coated baicalin significantly reduced MDA levels and elevated CAT, GSH-Px and T-AOC collectively enhancing their holistic antioxidant capacity.

Systemic health critically determines animal growth quality. Pro-inflammatory and anti-inflammatory cytokine balances regulate inflammation while maintaining homeostasis [32]. Key biomarkers include anti-inflammatory IL-4 and IL-10 and pro-inflammatory TNF-α, IL-1, and IL-6 [33, 34]. Previous studies have indicated that dietary baicalin attenuated systemic inflammation in mice by suppressing pro-inflammatory mediators through inhibition of NF-κB signaling and NLRP3 inflammasome activation [35]. Furthermore, baicalin downregulated pro-inflammatory cytokines (TNF-α, IL-1β, IL-6) and upregulated type I (IFN-α/β) and III (IFN-λ) interferons in neutrophils, attenuating inflammation [36]. IL-10 plays an essential role in intestinal homeostasis since IL-10 knockout mice serve as established genetic models for inflammatory bowel disease (IBD) research [37, 38]. In mouse models, baicalin lessened the severity of polymicrobial sepsis by raising IL-10 levels and dramatically lowering plasma and peritoneal fluid levels of IL-6, TNF-α, and IL-17A [39]. In Hu sheep, we found that both baicalin and coated baicalin reduced serum TNF-α/IL-6 and increased IL-4 levels. While both BAI and C-BAI increased serum IgG and IgM, only the C-BAI group exhibited elevated IgA and IL-10. Given the roles of IgA in mucosal immunity and IL-10 in anti-inflammation [40], these highlight the superior efficacy of the enteric-coated formulation in promoting intestinal health via targeted delivery. Additionally, both the BAI and C-BAI groups exhibited increased feed intake which likely contributed to improved systemic metabolism [41]. The C-BAI group demonstrated significantly greater improvements in immune-related indicators that included immunoglobulins and interleukins. Since feed intake was comparable between the two treatment groups, these differential effects suggested that the efficacy of the coated formulation was not merely a secondary consequence of enhanced nutrition. Instead, the data indicated that enteric coating potentiates the bioactivity of baicalin through targeted intestinal delivery and protection from ruminal degradation.

Bacteroidota and Firmicutes dominated the rumen microbiome in Hu sheep, consistent with ruminant studies [42, 43]. The Bacteroidota degrade non-structural carbohydrates [44] while Firmicutes ferment structural polysaccharides producing acetate and propionate [45]. These are the primary VFAs driving rumen fermentation. Acetate is derived from fibrolytic fermentation and is the major precursor for milk fat synthesis while propionate drives hepatic gluconeogenesis to support lipogenesis and lactose production. The A/P ratio reflects energy efficiency. The BAI group increased ruminal propionate by 44.6% and reduced the A/P ratio by 27.6% compared with CON, suggesting a shift toward propionate-type fermentation. These changes were associated with greater relative abundance of *Succinivibrio* (*P* < 0.05) and *Acetitomaculum* (*P* < 0.01). Prior studies have demonstrated that *Succinivibrio* competes with methanogens for H₂, producing succinate and suppressing methanogenesis [46]. This succinate is converted to propionate via microbial pathways while *Acetitomaculum* is a key ruminal acetogen and contributes to VFA synthesis [47, 48]. In Hu sheep, we found that baicalin supplementation corresponded to elevated propionate concentrations and increased abundance of *Succinivibrio*. The observed shift toward propionate-type fermentation was accompanied by enhanced VFA synthesis and improved growth performance.

Through the analysis of α diversity of rumen and jejunum flora revealed that C-BAI group reduced jejunal microbiota richness whereas the BAI group displayed no significant effect on ruminal diversity. This differential impact might pertain to C-BAI stimulation of bile acid secretion. *S. baicalensis* administration modulates gut microbiota in diabetic rats and suppressed the intestinal farnesoid X receptor (FXR) expression while enhancing hepatic cholesterol 7α-hydroxylase (CYP7A1) activation [49]. This process promoted hepatic cholesterol-to-bile acid conversion, increasing luminal bile acid levels. Similarly, naringin (a flavonoid found in grapefruit), elevated bile salt hydrolase (BSH)-producing and 7α-dehydroxylating microbiota in atherosclerotic mice while inhibiting the FXR/FGF15 pathway. This thereby upregulating 7α-dehydroxylase expression and accelerated cholesterol-to-bile acid conversion [50]. In the current work, jejunal metabolomics indicated elevated isoursodeoxycholic acid levels in the C-BAI group compared with the CON group (Table S2). This elevation correlates with baicalin-induced microbial restructuring and supported by KEGG enrichment of secondary bile acid biosynthesis pathways (Fig. S1) and elevated taurochenodeoxycholate levels in C-BAI (Table S3). Baicalin from the uncoated formulation was primarily metabolized in the rumen, which might have limited its jejunal bioavailability and subsequent bile acid stimulation. However, in the C-BAI group, increased jejunal Proteobacteria abundance was observed and most likely reflected enhanced intestinal exposure to bioactive baicalin. While this phylum encodes carbohydrate-active enzymes (CAZymes) that ferment dietary polysaccharides, elevated Proteobacteria levels are typically associated with gut dysbiosis and inflammation [51], However, C-BAI supplementation induced no inflammation and significantly elevated circulating anti-inflammatory mediators. This likely stems from baicalin's dual capacity to enhance systemic immunocompetence and neutralize Proteobacteria pathogenicity. Both baicalin and coated baicalin exerted effects in the jejunum but yielded divergent outcomes. This discrepancy likely arose from formulation-dependent pharmacokinetics underscoring the need for further investigation into site-specific delivery thresholds. As a genus within the Firmicutes phylum, *Lachnoclostridium* contributes significantly to polysaccharide hydrolysis and essential amino acid biosynthesis within the rumen microbial community [42]. This genus could enhance intestinal barrier integrity in mice and demonstrates protective effects against experimental colitis [52]. *Treponema* has been associated with adverse health effects in ruminants including disruption of iron homeostasis, induction of systemic inflammation and oxidative stress [53]. Dermatitis-associated treponemes have been identified at frequencies of up to 87% in affected lesions [54] and this genus is further linked to infectious hoof lesions in ruminants [55]. *Ralstonia* is a low-virulence, Gram-negative, non-fermentative bacterium and recent reports indicated an increased incidence of *Ralstonia*-associated infections that also exhibited broad-spectrum antibiotic resistance [56]. In our study, baicalin significantly reduced the relative abundance of *Ralstonia* in the rumen, suggesting its potential as a dietary phytobiotic to mitigate infections caused by multidrug-resistant *Ralstonia* strains. Conversely, the physiological role of *Bradyrhizobium* in ruminants remains poorly characterized. In the preputial microbiota of healthy bulls, *Bradyrhizobium* has been detected exclusively in low-diversity microbial communities suggesting niche specialization in microbially simplified environments [57]. Consistent with this observation, we found that jejunal bacterial diversity was decreased in Hu sheep receiving coated baicalin, although the underlying mechanisms require further investigation. In the C-BAI group, decreased *Eubacterium coprostanoligenes* abundance was associated with increased jejunal levels of taurochenodeoxycholate and isodeoxycholic acid. This association might indicate a shift in cholesterol utilization toward bile salt synthesis that in turn affect *E. coprostanoligenes* proliferation. However, a causal relationship remains to be established. Although both BAI and C-BAI modulated the jejunal microbiota composition and coated baicalin specifically enriched microbial populations associated with insoluble polysaccharide degradation and soluble protein assimilation, thereby improving nutrient utilization efficiency.

Metabolomics provides a comprehensive explanation of phenotypic alterations compared to genomics or proteomics [58]. Multiple studies have shown a strong correlation between rumen and intestinal microbes and metabolites [21, 59]. Our results revealed significant changes in the levels of numerous ruminal and jejunal metabolites in both the BAI and C-BAI groups. These were likely mediated by shifts in microbiota abundance within these gastrointestinal regions. Notably, the BAI group had increased accumulation of health-promoting metabolites in the rumen, whereas the C-BAI group showed preferential enrichment of growth-enhancing metabolites in the jejunum. Substantially elevated levels of propionate, butyrate, isovalerate, linolenic acid, homogentisic acid and 2,3-diphospho-D-glycerate were detected in the rumen of the BAI group. The increased levels of propionate, butyrate and isovalerate were consistent with our ruminal fermentation metrics and indicated that baicalin stimulates microbially derived VFA production that in turn, enhanced the growth and development in the sheep. Linolenic acid is an essential fatty acid in animals, comprising two isomers: α-linolenic acid and γ-linolenic acid. α-Linolenic acid exerts beneficial effects on improving ruminal microbial fermentation and increasing total VFA levels [60]. Homogentisic acid (2,5-dihydroxyphenylacetic acid) is a metabolic intermediate in the phenylalanine-tyrosine catabolic pathway that exhibits significant antioxidant and free radical-scavenging activities. Furthermore, it protects against the depletion of polyunsaturated fatty acids and cholesterol while preventing the accumulation of their oxidized derivatives including MDA [61]. Consequently, the elevated homogentisic acid concentration observed in the BAI group may augment systemic antioxidant capacity in Hu sheep. The C-BAI group exhibited higher jejunal levels of D-2-phosphoglycerate and linoleic acid. Within the glycolytic pathway, D-2-phosphoglycerate undergoes enzyme-catalyzed dehydration to phosphoenolpyruvate via α-enolase whereas phosphoenolpyruvate is subsequently converted to pyruvate by pyruvate kinase with concomitant substrate-level phosphorylation of ADP to ATP [62, 63]. ATP supplies energy for bodily functions and pyruvate is converted to glucose via the gluconeogenesis pathway. Linoleoylglycine is an endogenous anti-inflammatory lipid that facilitates chronic inflammation regression [64]. Integrated microbiome and metabolome analyses revealed that both baicalin and coated baicalin modulated microbial communities in the rumen and jejunum, promoted beneficial taxa associated with enhanced production of these types of functional metabolites. These shifts contributed to improved growth performance in Hu sheep.

## Conclusions

Dietary supplementation with baicalin or coated baicalin in fattening Hu sheep promoted the production of VFA and other beneficial metabolites via regulation of the crosstalk between gastrointestinal microbiota and metabolites while suppressing potential pathogenic flora. These effects were associated with enhancements in antioxidant capacity and immune function and contributed to improved growth performance. Although coated baicalin exhibited advantages in certain immune parameters, uncoated baicalin demonstrated superior effects on growth performance, particularly feed conversion efficiency. Overall, these results support the potential of baicalin as a promising dietary supplement for improving health and production efficiency under the conditions of this study.

## Supplementary Information

Additional file 1: Table S1. Differential metabolites in the rumen of CON and BAI group animals (Top 50). Table S2. Differential metabolites in jejunum of CON and C-BAI group animals (41 in total). Table S3. Differential metabolites in jejunum of BAI and C-BAI group animals. Table S4. Pathway information related to differential metabolites in the rumen of CON and BAI groups (Top 10). Table S5. Pathway information related to differential metabolites in the rumen of CON and C- BAI group animals (Top 10). Fig. S1. Metabolic pathway analysis of jejunum differential metabolites in the BAI and C-BAI groups.

## Data Availability

The sources of all the materials used in this article have been clearly stated. The datasets supporting the conclusions of this article can be made available on reasonable request to the corresponding author.

## Abbreviations

ADF: Acid detergent fiber
ADG: Average daily gain
ANOVA: Analysis of variance
A/P: The ratio of acetic/propionic
CP: Crude protein
CAT: Catalase
DMI: Dry matter intake
EE: Ether extract
FBW: Final body weight
F/G: Feed conversion ratio
GSH-Px: Glutathione peroxidase
IBW: Initial body weight
IgA: Immunoglobulin A
IgG: Immunoglobulin G
IgM: Immunoglobulin M
IFN-γ: Interferon-γ
IL-1: Interleukin-1
IL-4: Interleukin-4
IL-6: Interleukin-6
IL-10: Interleukin-10
KEGG: Kyoto Encyclopedia of Genes and Genomes
ME: Metabolizable energy
MDA: Malondialdehyde
NDF: Neutral detergent fiber
NH_3_-N: Ammonia nitrogen
OM: Organic matter
OTUs: Operational taxonomic units
PLS-DA: Partial least squares discriminant analysis
TWG: Total weight gain
T-AOC: Total antioxidant capacity
TNF-α: Tumour necrosis factor-α
VFA: Volatile fatty acid
