# Supplementary material for: Dietary baicalin supplementation enhances growth performance in fattening Hu sheep via dual modulation of immunity and gastrointestinal microbiome-metabolic crosstalk
Source: J Anim Sci Biotechnol. 2026 May 30;17:104. doi: 10.1186/s40104-026-01413-y (PMC13221762; doi:10.1186/s40104-026-01413-y)
Supplement: Supplementary file 1 — Additional file 1: Table S1. Differential metabolites in the rumen of CON and BAI group animals (Top 50). Table S2. Differential metabolites in jejunum of CON and C-BAI group animals (41 in total). Table S3. Differential metabolites in jejunum of BAI and C-BAI group animals. Table S4. Pathway information related to differential metabolites in the rumen of CON and BAI groups (Top 10). Table S5. Pathway information related to differential metabolites in the rumen of CON and C- BAI group animals (Top 10). Fig. S1. Metabolic pathway analysis of jejunum differential metabolites in the BAI and C-BAI groups. [file 40104_2026_1413_MOESM1_ESM.docx]

**Table S1** Differential metabolites in the rumens of CON and BAI group animals (Top 50)

| **Metabolites** | **VIP1** | **log_2__FC2** | ***P*-value** |
| --- | --- | --- | --- |
| Saccharin | 20.85 | 0.65 | 0.036 |
| Butanoic acid | 20.25 | 1.03 | 0.006 |
| Isovaleric aci | 17.13 | 0.57 | 0.015 |
| D-mannosamine | 14.61 | 0.98 | 0.034 |
| Halofenozid | 11.05 | 1.60 | 0.007 |
| 21-Hydroxypregnenolon | 9.90 | 2.07 | 0.005 |
| Schweinfurthine | 7.61 | 2.61 | 0.035 |
| Propionic acid | 7.04 | 0.92 | 0.014 |
| Phenylacetic acid + 2o, o-hex | 6.80 | 0.98 | 0.012 |
| 3,4-dihydroxyhydrocinnamic acid | 5.20 | 1.00 | 0.023 |
| 3-hydroxykynurenin | 4.95 | 1.97 | 0.010 |
| 4-hydroxyquinoline | 4.88 | 1.41 | 0.032 |
| Nonanoic acid | 4.81 | 0.55 | 0.033 |
| Estrone | 4.63 | 0.49 | 0.038 |
| 21-hydroxyprogesterone | 4.58 | 1.73 | 0.005 |
| Methyl hexadecanoate | 4.42 | 0.87 | 0.008 |
| 2-oleoyl-1-palmitoyl-sn-glycero-3-phosphocholine | 4.40 | 0.61 | 0.013 |
| N-lauroyl-d-erythro-sphingosylphosphorylcholine | 4.35 | 1.32 | 0.003 |
| Linolenic acid | 3.99 | 1.73 | 0.006 |
| Putrescine | 3.95 | 1.71 | 0.011 |
| Formononeti | 3.55 | 1.23 | 0.022 |
| 2,3-diphospho-d-glyceric acid | 3.48 | 0.63 | 0.038 |
| 3-acetylindole | 3.44 | 1.35 | 0.006 |
| β-amyrin acetate | 3.06 | 1.14 | 0.040 |
| 4-hydroxy-5e,7z,10z,13z,16z,19z-docosahexaenoic acid | 3.00 | 2.46 | 0.002 |
| Tetrahydrocorticosterone | 2.98 | 1.90 | 0.004 |

Continuation of Schedule 1

| **Metabolites** | **VIP1** | **log_2__FC2** | ***P*-value** |
| --- | --- | --- | --- |
| 2-(2',3',4'-trihydroxybutyl) quinoxaline | 2.84 | 0.58 | 0.009 |
| 1h-pyrano [3',4':6,7] indolizino [1,2-b] quinoline-3,14(4h,12h)-dione, 4,11-diethyl-4,9-dihydroxy-, (4s)- | 2.81 | 0.81 | 0.037 |
| Evodin | 2.60 | 2.80 | 0.012 |
| Prostaglandin e1 | 2.59 | 1.06 | 0.006 |
| Pleiomutine | 2.45 | 9.34 | 0.049 |
| Sinomenine | 2.44 | 0.76 | 0.038 |
| Schizandrina | 2.40 | 1.19 | 0.048 |
| Indica | 2.39 | 1.70 | 0.013 |
| Pinolidoxin | 2.29 | 2.03 | 0.006 |
| Thifluzamide | 2.25 | 1.11 | 0.031 |
| Lys-Gly | 2.21 | 0.49 | 0.010 |
| N-acetylputrescine | 2.20 | 1.47 | 0.021 |
| 4-hydroxyphenethyl alcohol | 2.19 | 1.09 | 0.022 |
| 1-palmitoylglycerol | 2.15 | 1.09 | 0.003 |
| Pyruvaldehyde | 2.12 | 1.33 | 0.050 |
| Imatinib | 2.11 | 8.11 | 0.049 |
| Sm d30:1 | 2.07 | 1.40 | 0.025 |
| Homogentisic acid | 1.99 | 1.24 | 0.037 |
| Albizziin | 1.95 | 1.84 | 0.014 |
| Oxadixyl | 1.91 | 1.35 | 0.003 |
| Simazine | 1.86 | 0.47 | 0.022 |
| Methanone, [1-(5-fluoropentyl)-6-hydroxy-1h-indol-3-yl] (2,2,3,3-tetramethylcyclopropyl)- | 1.83 | 2.61 | 0.002 |
| 2(1h)-pyridinone | 1.76 | 0.96 | 0.028 |
| Ketamine | 1.72 | 0.78 | 0.026 |
| Vincamone | 1.68 | 0.96 | 0.001 |

*VIP* Variable importance in projection

**Table S2** Differential metabolites in jejunum of CON and C-BAI group animals (41 in total)

| **Metabolites** | **VIP** | **log_2__FC** | ***P*-value** |
| --- | --- | --- | --- |
| 1-oleoyl-sn-glycero-3-phosphocholine | 6.97 | 0.88 | 0.009 |
| Stearoyl-l-carnitine | 6.67 | 1.22 | 0.027 |
| Securinine | 6.44 | 1.05 | 0.013 |
| 3-hydroxykynurenine | 4.74 | 2.15 | 0.044 |
| Linoleoylglycine | 4.70 | 1.52 | 0.018 |
| N-oleoylglycine | 3.92 | 1.73 | 0.020 |
| 4-hydroxyphenylmaraviroc | 3.88 | 1.00 | 0.050 |
| Anisatin | 3.17 | 0.88 | 0.045 |
| D-pyroglutamic acid | 3.13 | 0.23 | 0.021 |
| D-glutamine | 3.07 | 0.22 | 0.020 |
| Ilimaquinone | 2.81 | 0.56 | 0.046 |
| Prostaglandin e2 | 2.79 | 1.36 | 0.026 |
| 1-(1,2-dihexadecanoylphosphatidyl) inositol-3-phosphate | 2.64 | 1.74 | 0.033 |
| 3,4-Dihydroxy-L-phenylalanine(L-DOPA) | 2.49 | 1.05 | 0.048 |
| Hydroquinidine | 2.43 | 0.23 | 0.027 |
| Thymol-beta-d-glucoside | 2.37 | 0.25 | 0.028 |
| (+)-6-aminopenicillanic acid | 2.16 | 1.72 | 0.026 |
| D-2-phosphoglyceric acid | 2.09 | 0.62 | 0.005 |
| 11beta-hydroxyprogesterone | 2.02 | 0.84 | 0.029 |
| 8-(2-hydroxy-3-methoxy-3-methylbutyl)-7-methoxychromen-2-one | 2.01 | 0.81 | 0.000 |
| Betulinic acid | 1.89 | 3.57 | 0.050 |
| Benzalkonium chloride | 1.76 | 2.49 | 0.027 |
| Gly-Lys | 1.70 | 1.39 | 0.023 |
| 21-Hydroxypregnenolone | 1.53 | 0.98 | 0.029 |
| Ostruthi | 1.47 | 0.32 | 0.015 |
| 13-hpode13 | 1.42 | 0.77 | 0.012 |
| 1-(4-isopropylphenyl)-3-methylurea | 1.42 | 0.75 | 0.038 |
| Phosphatidylcholine lyso 20:4 | 1.42 | 1.30 | 0.037 |
|  |  |  |  |

Continuation of Schedule 2

| **Metabolites** | **VIP** | **log_2__FC** | ***P*-value** |
| --- | --- | --- | --- |
| (2-aminoethoxy) [3-[hexadec-1-en-1-yloxy]-2-[icosa-5.8.11.14-tetraenoyloxy] propoxy] phosphinic acid | 1.34 | 1.97 | 0.046 |
| Hydrocinnamic acid | 1.22 | 0.89 | 0.006 |
| Mucic acid | 1.22 | 1.17 | 0.037 |
| Acridine orange | 1.19 | 1.25 | 0.040 |
| Fahfa 26:0 | 1.15 | 2.38 | 0.037 |
| 3,5-di-tert-butyl-4-hydroxybenzoic acid | 1.14 | 0.60 | 0.019 |
| (-)-caryophyllene oxide | 1.14 | 2.32 | 0.022 |
| 2-linoleoylglycerol | 1.14 | 1.22 | 0.018 |
| Isodeoxycholic acid | 1.09 | 1.30 | 0.033 |
| beta.-tocotrienol | 1.07 | 1.89 | 0.000 |
| Osajin | 1.05 | 1.08 | 0.045 |
| Antipain | 1.05 | 1.00 | 0.009 |
| (6r)-2-(hydroxymethyl)-6-((3r,5r,7r,8r,9s,10s,12s,13r,14s,17r)-3,7,12-trihydroxy-10,13-dimethylhexadecahydro-1h-cyclopenta[a]phenanthren-17-yl) heptanoic acid | 1.03 | 0.77 | 0.030 |

*VIP* Variable importance in projection

**Table S3** Differential metabolites in jejuni of BAI and C-BAI group animals

| **Metabolites** | **VIP** | **log_2__FC** | ***P*-value** |
| --- | --- | --- | --- |
| Taurochenodeoxycholate | 56.39 | 0.59 | 0.032 |
| Isethionate | 3.96 | 0.59 | 0.034 |
| Oleoyl-l-carnitine | 3.60 | 0.49 | 0.031 |
| Glutamate conjugated cholic acid | 3.55 | 0.65 | 0.035 |
| 7-methylguanine | 3.48 | 0.18 | 0.002 |
| DL-leucine | 2.5 | 1.10 | 0.037 |
| (6r)-2-hydroxy-2-methyl-6-((3r,5s,7r,8r,9s,10s,12s,13r,14s,17r)-3,7,12-trihydroxy-10,13-dimethylhexadecahydro-1h-cyclopenta[a]phenanthren-17-yl) heptyl hydrogen sulfate | 2.07 | 0.35 | 0.045 |
| Prostaglandin d1 | 1.99 | 0.39 | 0.036 |
| N-lauroyl-d-erythro-sphingosylphosphorylcholine | 1.86 | 2.13 | 0.029 |
| L-alaninamide, n-[2-[2-(hydroxyamino)-2-oxoethyl]-4-methyl-1-oxopentyl]-3-(2-naphthalenyl)-l-alanyl- | 1.85 | 1.17 | 0.029 |
| 1-methylxanthine | 1.79 | 0.96 | 0.040 |
| Ser-His-Lys | 1.75 | 0.37 | 0.019 |
| Decanoyl-l-carnitine | 1.68 | 0.82 | 0.037 |
| Fendilin | 1.63 | 0.82 | 0.015 |
| 3-oxazolidinecarboxylic acid, 2,2-dimethyl-4-(1-oxo-2-hexadecyn-1-yl)-, 1,1-dimethylethyl ester, (4s)- | 1.38 | 1.00 | 0.008 |
| Pg 30:0 | 1.35 | 3.45 | 0.019 |
| Leucine | 1.32 | 0.75 | 0.005 |
| C10-dats | 1.20 | 1.18 | 0.048 |
| Suberic acid | 1.18 | 1.17 | 0.024 |
| Cys-Arg | 1.16 | 0.74 | 0.048 |
| Prostaglandin f2. alpha. 1,9-lactone | 1.14 | 0.53 | 0.024 |
| (9s,10r,13r,14s,17r)-17-((r)-5-hydroxypentan-2-yl)-10,13-dimethyl-1,2,6,7,8,9,10,11,12,13,14,15,16,17-tetradecahydro-3h-cyclopenta[a]phenanthren-3-one | 1.08 | 0.53 | 0.036 |

*VIP* Variable importance in projection

**Table S4** Pathway information related to differential metabolites in the rumen of CON and BAI groups (Top 10)

| **Pathway** | **Total** | **Hits** | ***P*-value** |
| --- | --- | --- | --- |
| Propanoate metabolism | 9 | 2 | 0.023 |
| Tyrosine metabolism | 24 | 3 | 0.025 |
| Ethylbenzene degradation | 2 | 1 | 0.054 |
| Arginine and proline metabolism | 16 | 2 | 0.068 |
| Biosynthesis of alkaloids derived from histidine and purine | 16 | 2 | 0.068 |
| Furfural degradation | 3 | 1 | 0.080 |
| Styrene degradation | 3 | 1 | 0.080 |
| Progesterone, androgen and estrogen receptor agonists/antagonists | 4 | 1 | 0.106 |
| Inositol phosphate metabolism | 5 | 1 | 0.130 |
| Purine metabolism | 25 | 2 | 0.147 |

**Table S5** Pathway information related to differential metabolites in the rumen of CON and C- BAI group animals (Top 10)

| **Pathway** | **Total** | **Hits** | ***P*-value** |
| --- | --- | --- | --- |
| Penicillins | 1 | 1 | 0.02 |
| Cephalosporins - parenteral agents | 1 | 1 | 0.02 |
| Glycerolipid metabolism | 3 | 1 | 0.04 |
| Penicillin and cephalosporin biosynthesis | 4 | 1 | 0.06 |
| Linoleic acid metabolism | 5 | 1 | 0.07 |
| Biosynthesis of plant secondary metabolites | 69 | 3 | 0.08 |
| Glycolysis / Gluconeogenesis | 8 | 1 | 0.12 |
| Ascorbate and aldarate metabolism | 9 | 1 | 0.13 |
| Pentose phosphate pathway | 11 | 1 | 0.15 |
| Biosynthesis of alkaloids derived from shikimate pathway | 50 | 2 | 0.17 |

**Figure**


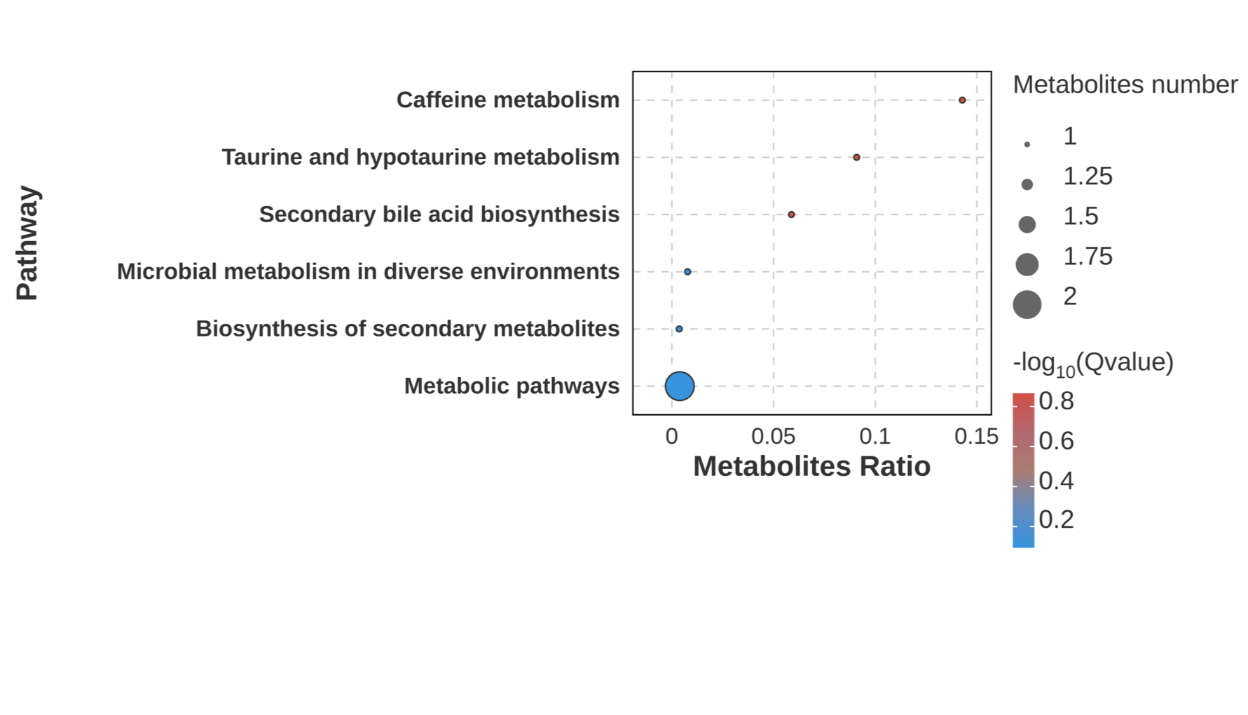


**Fig. S1** Metabolic pathway analysis of jejunum differential metabolites in the BAI and C-BAI groups
